# A new species of *Liolaemus* related to *L. nigroviridis* from the Andean highlands of Central Chile (Iguania, Liolaemidae)

**DOI:** 10.3897/zookeys.555.6011

**Published:** 2016-01-20

**Authors:** Jaime Troncoso-Palacios, Alvaro A. Elorza, German I. Puas, Edmundo Alfaro-Pardo

**Affiliations:** 1 Programa de Fisiologia y Biofisica, Instituto de Ciencias Biomedicas (ICBM), Facultad de Medicina, Universidad de Chile, Independencia 1027, Santiago, Chile; 2 Centro de Investigaciones Biomedicas, Facultad de Ciencias Biologicas y Facultad de Medicina, Universidad Andres Bello, Republica 239, Santiago, Chile; 3 Instituto Milenio de Inmunologia e Inmunoterapia, Portugal 49, Santiago, Chile; 4 Gayana Ecolodge, Cadillal Km 7, Corral, Chile

**Keywords:** Liolaemus nigroviridis, Liolaemus uniformis sp. n., lizard, *Cyt-b*, mtDNA

## Abstract

The *Liolaemus nigroviridis* group is a clade of highland lizards endemic to Chile. These species are distributed from northern to central Chile, and currently there are no cases of sympatric distribution. This study describes a new species, *Liolaemus uniformis* **sp. n.**, from this group, and provides a detailed morphological characterization and mitochondrial phylogeny using cytochrome-b. *Liolaemus uniformis* was found in sympatry with *Liolaemus nigroviridis* but noticeably differed in size, scalation, and markedly in the color pattern, without sexual dichromatism. This new species has probably been confused with *Liolaemus monticola* and *Liolaemus bellii*, both of which do not belong to the *nigroviridis* group. The taxonomic issues of this group that remain uncertain are also discussed.

## Introduction

The *Liolaemus nigroviridis* group is a clade of highland lizards endemic to central and northern Chile, the species of which are allopatrically distributed (Pincheira-Donoso and Núñez 2005). Almost all species of this group have a complicated taxonomic history with several cases of synonymies (*e.g*. Núñez and Jaksic 1992, Pincheira-Donoso and Núñez 2007, Troncoso-Palacios 2013). In his book on lizards from northwest, northeast, and eastern Argentina, Cei (1993) proposed the *nigroviridis* group and included within it *Liolaemus constanzae* Donoso-Barros, 1961. Lobo (2001) performed the first cladistic analysis of this group and, based on morphological characteristics, included the following species: *Liolaemus campanae* Hellmich, 1950, *Liolaemus lorenzmuelleri* Hellmich, 1950, *Liolaemus maldonadae* Núñez, Navarro & Loyola, 1991, and *Liolaemus nigroviridis* Müller & Hellmich, 1932. Later, Lobo (2005), updating the morphological phylogeny, added to the *nigroviridis* group *Liolaemus nigroroseus* Donoso-Barros, 1966 and *Liolaemus isabelae* Navarro & Núñez, 1993, but excluded *Liolaemus lorenzmuelleri*.

Pincheira-Donoso and Núñez (2005), through phenetic analysis recovered all of the species listed by Lobo (2005) within the *nigroviridis* group and also reincorporated *Liolaemus lorenzmuelleri* and *Liolaemus constanzae*. Furthermore, *Liolaemus constanzae* was listed with two subspecies, *Liolaemus constanzae constanzae* and *Liolaemus constanzae donosoi* Ortiz, 1975. These authors also incorporated *Liolaemus juanortizi* Young-Downey & Moreno, 1991 and *Liolaemus melanopleurus* (Philippi, 1860), the latter of which was included as *incertae sedis*. Moreover, *Liolaemus nigroroseus* was considered a junior synonym of *Liolaemus constanzae*, as has also been proposed by other authors (Núñez and Jaksic 1992, Troncoso-Palacios 2013), while *Liolaemus campanae* was regarded as a junior synonym of *Liolaemus nigroviridis*. In fact, *Liolaemus campanae* was previously described as a subspecies of *Liolaemus nigroviridis* (Hellmich 1950) and later proposed to be a synonym of *Liolaemus nigroviridis* (Núñez and Jaksic 1992, Valencia et al. 1979).

Lobo et al. (2010) accepted all of the species listed by Pincheira-Donoso and Núñez (2005) as members of the *nigroviridis* group, except for *Liolaemus donosoi* which they placed into the *nigromaculatus* group. Finally, Troncoso-Palacios (2013) indicated that *Liolaemus donosoi* is a junior synonym of *Liolaemus constanzae*, as previously suggested (Núñez and Jaksic 1992, Veloso et al. 1982), and recognized the seven species listed by Lobo et al. (2010) as members of the *nigroviridis* group – *Liolaemus constanzae*, *Liolaemus isabelae*, *Liolaemus juanortizi*, *Liolaemus lorenzmuelleri*, *Liolaemus maldonadae*, *Liolaemus melanopleurus*, and *Liolaemus nigroviridis*.

Very few studies have used molecular data within this group. Schulte and Moreno-Roark (2010) constructed a mitochondrial phylogeny of 733 Iguanian lizards. The authors concluded that *Liolaemus nigroviridis nigroviridis*, and *Liolaemus nigroviridis campanae* are sister taxa and that *Liolaemus isabelae* does not belong to the *nigroviridis* group. Cianferoni et al. (2013) performed a cytochrome-b (*Cyt-b*) phylogeographic study in *Liolaemus nigroviridis* populations and proposed that this species could contain at least two different species-level lineages.

In a field trip to the vicinity of Piuquenes (Valparaíso Region, Chile), we believe we found some populations probably previously assigned to *Liolaemus monticola* Müller & Hellmich, 1932 by Núñez et al. (2010:57). Subsequent *Cyt-b* phylogenetic analysis and morphological comparisons determined that this population represents a new species that belongs to the *nigroviridis* group. This new species occurred in sympatry with *Liolaemus nigroviridis*, constituting the first case of sympatry within this group of lizards.

The current study describes this new species and provides a full diagnosis in regards to other species of the *nigroviridis* group. Although the color pattern of this new species resembles *Liolaemus juanortizi* and *Liolaemus lorenzmuelleri*, the scalation is markedly different and the distribution is allopatric (> 240 km of separation). Moreover, various taxonomical aspects of the *nigroviridis* group that require attention are discussed.

## Materials and methods

### Morphological data and analyses

Specimens of all species currently considered within the *nigroviridis* group were examined. Morphological characteristics were examined according to Etheridge (1995) and Lobo (2005). Body measurements were taken with a digital Vernier caliper (0.02 mm precision) and given as the mean ± standard deviation (x ± SD). We applied a Kolmogorov-Smirnov test to verify data normality, a subsequent t-test or Mann-Whitney U test was used if data passed or failed the normality test, respectively, to compare scale count (midbody, dorsal and ventral) and size (snout vent length, SVL) of the new species against some related species (*Liolaemus constanzae*, *Liolaemus juanortizi*, *Liolaemus lorenzmuelleri* and *Liolaemus nigroviridis*). Only significant results are presented. Scales were observed with different magnifying lenses. Scalation and measurements were recorded on the right side of the specimen. Dorsal scales were counted between the occiput and the anterior border of the hind limbs. Ventral scales were counted from the mental scale to the anterior margin of the cloacal opening. Stomach and intestinal contents were analyzed under a binocular stereoscope for one specimen of the new species. Data for the midbody scales of *Liolaemus juanortizi* were taken from one revised specimen and six reported in Young-Downey and Moreno (1991). Classification was carried out considering species currently assigned to the *nigroviridis* group (Troncoso-Palacios 2013). *Liolaemus isabelae* is included in the comparison but the relationship of this species with the *nigroviridis* group is uncertain (see Discussion). The examined specimens are listed in Appendix I. Some mapping data were taken from existing literature or field observations without specimen collection: 1) *Liolaemus nigroviridis* from Manque (Mella 2005), El Arpa and El Roble (Cianferoni et al. 2013), Riecillo (Núñez et al. 2010), Campana (Hellmich 1050), Chepical and Juncal (field observations, 32°16'S - 70°30'W and 32°53'S - 70°07'W respectively); 2) *Liolaemus maldonadae* from Los Molles (Núñez et al. 1991). Acronyms used are: Museo Nacional de Historia Natural de Chile (MNHNCL), Museo de Zoología de la Universidad de Concepción (MZUC) and Colección de Flora y Fauna, Profesor Patricio Sánchez Reyes of the Pontificia Universidad Católica de Chile (SSUC).

### DNA purification, PCR amplification, and sequencing

Samples from liver and thigh muscle were obtained from ethanol-fixed lizards which were subject to a rehydration process according to Coura (2005). Samples were washed twice in distilled water for 5 min at 55 °C to remove the fixative and then rehydrated with 1x Tris/EDTA for 5 min at 55 °C and then 1M Tris pH 7.5, at 55 °C overnight. Right after, samples were digested with proteinase K (20 mg/ml) at 55 °C overnight. Genomic DNA isolation (mitochondrial and nuclear) was done with the Wizard® Genomic DNA Purification kit (Cat # A1120, Promega, USA) following manufacturer´s instructions. The mitochondrial gene *Cyt-b* was amplified from total DNA through two phase conventional PCR with the primers GLUDGL (5´-TGA CTT GAA RAA CCA YCG TTG-3´) and CB3 (5´-GGC AAA TAG GAA RTA TCA TTC-3´), reported in Torres-Pérez et al. (2009), to generate a 700bp amplicon. PCR reactions were performed with the SapphireAmp® Fast PCR Master Mix (Cat # RR350A, Takara Clontech, USA) using 100 ng of total genomic DNA as a template and following the instruction manual. Two-phase PCR cycling was as follows: Phase 1, initial 98 °C denaturation for 3 min, then 5 cycles of 98 °C denaturation for 30 s, 47 °C annealing for 45 s and 72 °C extension for 45 s. The Phase 2, next 40 cycles of 98 °C denaturation for 30 s, 58 °C annealing for 45 s and 72 °C extension for 45 s. A final 72 °C extension step for 5 min was added to finish the PCR. The 700 bp PCR amplicon was checked by DNA electrophoresis on a 1% agarose gel in 1x Tris-Acetate-EDTA (TAE) buffer. The amplicons were purified with the E.Z.N.A.® Cycle-Pure Kit (Cat # D6492-02, Omega Biotek, USA) and sent for capillary sequencing to Macrogen, Korea.

### Phylogenetic reconstruction

The accession numbers of the *Cyt-b* mitochondrial loci sequences generated in this study and the sequences obtained from GenBank are indicated in Appendix II. Forty three nucleotide sequences involved in the analysis were aligned using MUSCLE (Edgar 2004). We used the JModelTest v2.1.7 (Darriba et al. 2012, Guidon and Gascuel 2003) to select an appropriate substitution model (HKY + G + I), with a BIC index. We performed a Bayesian inference (BI) analyses with MrBayes v3.1.5 (Ronquist and Huelsenbeck 2003). Two independent analyses, each consisting of two groups of four chains that run independently, that were run for 15.0 × 10^6^ generation and a at sample frequency = 1000. Priors were let by default. *Phymaturus vociferator* Pincheira-Donoso 2004, was selected as the outgroup. The 25% of samples were discarded as burnin when calculating the convergence diagnostic, assessed examining values of average standard deviation of the Potential Scale Reduction Factor (PSRF) for all parameters.

## Results

**Figure 1.**
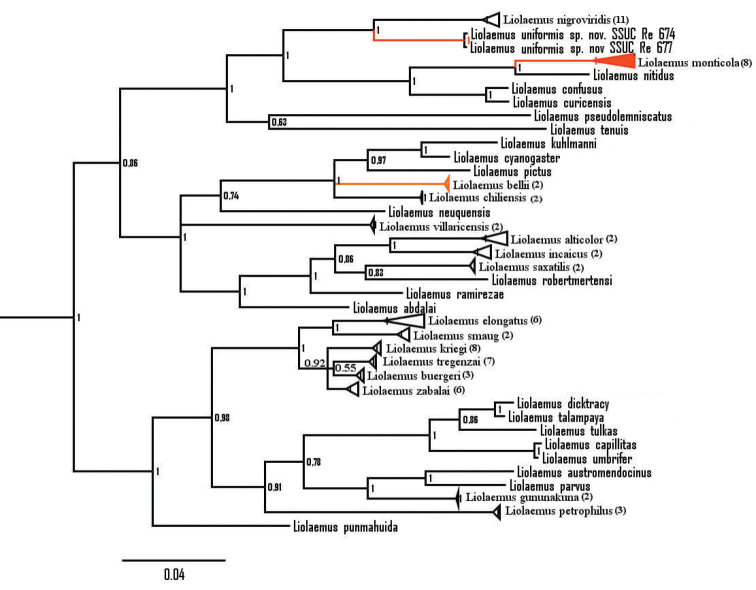
Bayesian inference of phylogeny tree using *Cyt-b* showing phylogenetic relationships of *Liolaemus uniformis* sp. n. (red) and related species (HKY+G+I model). *Liolaemus bellii* and *Liolaemus monticola*, probably confused with the new species, are also in red. Posterior probability is indicated at each node. Scale shows the number of substitutions per site. Number between parentheses indicates the number of sequences for the collapsed nodes.

The genetic tree constructed from mitochondrial DNA (mtDNA) (Fig. 1) placed the newly identified *Liolaemus* species as a sister taxon of *Liolaemus nigroviridis* (posterior probability pp = 1). However, no data are available for most of the species in the *nigroviridis* group as sample collection is hampered by the high altitudes where these species inhabit. Therefore, the discovered topology should be considered preliminary (see Discussion). *Liolaemus monticola* is nested with strong support (pp = 1) in the *monticola* group, the sister clade of the *nigroviridis* group. *Liolaemus bellii* is not closely related to the new *Liolaemus* or *Liolaemus monticola*, and is nested in a node with polytomy.

### Liolaemus uniformis sp. n.

Taxon classificationAnimaliaSquamataLiolaemidae

http://zoobank.org/B412BEF2-C337-4472-A4CE-9AFD73876B07

Fig. 2A, B

Liolaemus altissimus altissimus (in part?), Mella. 2005, Guía Camp. Rep. Chil. Zon. Cent., p. 38.
Liolaemus monticola ?, Núñez et al. 2010, Bol. Mus. Nac. Hist. Nat., p. 57.

#### Holotype.

SSUC Re 674. Adult male. Collected in the west shore of the Chepical Lagoon (32°15'S – 70°30'W), approximately 30 km NE Alicahue, San Felipe de Aconcagua Province, Valparaíso Region, Chile. Collectors: J. Troncoso-Palacios and E. Alfaro. December, 2012.

#### Paratypes

**Figure 2.**
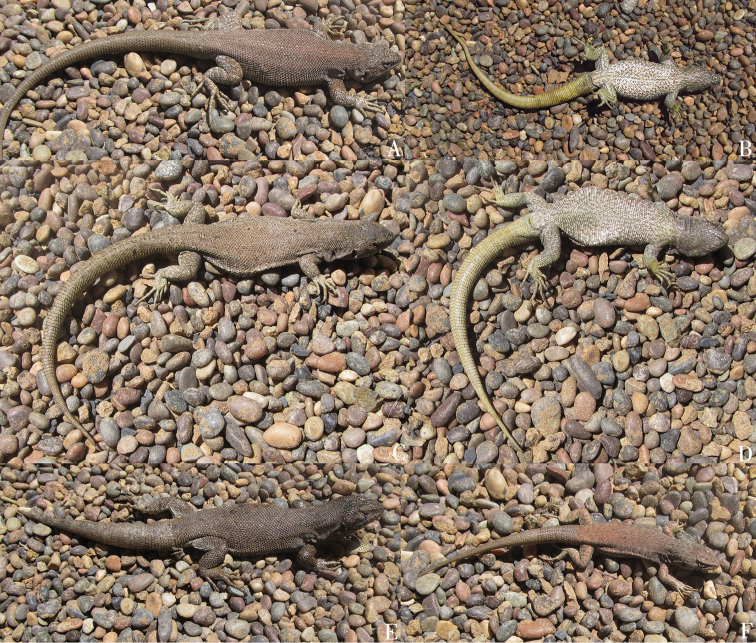
*Liolaemus uniformis* sp. n. **A, B** Holotype, male **C**, **D** Paratype, female **E** Paratype, male **F** Paratype, juvenile (unknown sex). All from the type locality.

(Fig. 2C, D, E, F). SSUC Re 675, male. SSUC Re 676–78, three females. SSUC Re 679, juvenile. The same data as the holotype.

#### Etymology.

The species name “*uniformis*” (Latin) refers to the lack of dorsal pattern and uniform color found for both males and females.

#### Diagnosis.

**Figure 3.**
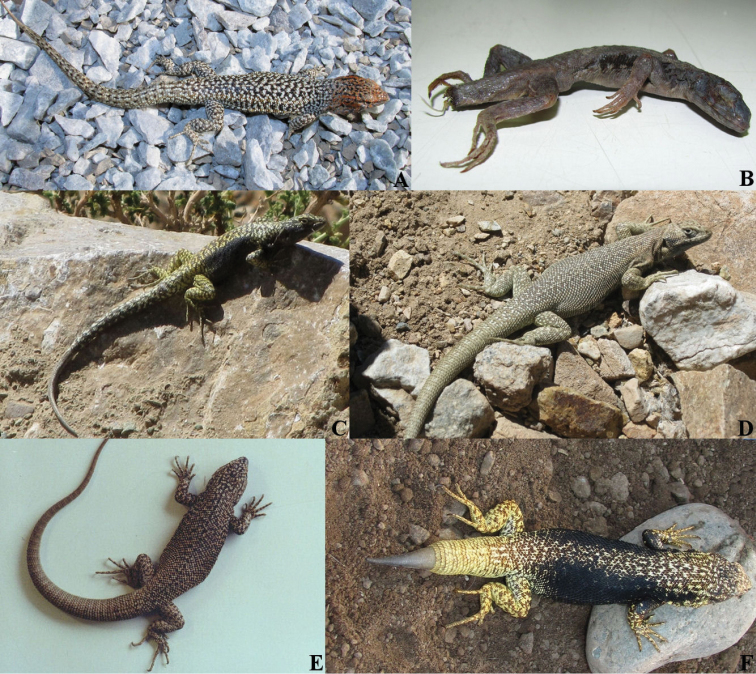
Chilean species of the *nigroviridis* group (with the exception of *Liolaemus nigroviridis*), ordered from north to south. **A** *Liolaemus constanzae*, male from vicinity of San Pedro (picture by JTP) **B** *Liolaemus melanopleurus*, male from Atacama (picture by JTP) **C** *Liolaemus isabelae*, male from Montandón (picture by JTP) **D** *Liolaemus juanortizi*, unknown sex specimen from road to Negro Francisco (picture by F. de Grotee) **E** *Liolaemus lorenzmuelleri*, unknown sex specimen from Embalse La Laguna (picture by A. Labra) **F** *Liolaemus maldonadae*, male from vicinity of Alcohuaz (picture by JTP).

*Liolaemus uniformis* is larger than *Liolaemus constanzae* (Mann–Whitney U = 0.5, P < 0.01, Table 1). *Liolaemus constanzae* has sexual dichromatism, a feature absent in *Liolaemus uniformis*. Males of *Liolaemus constanzae* have a black vertebral line and black spots on the paravertebral fields (Fig. 3A), whereas *Liolaemus uniformis* has no dorsal pattern. Additionally, the southern distributional limit of *Liolaemus constanzae* in Agua Verde, Antofagasta Region, Chile (Ortiz 1975), is more than 750 km north of the type locality recorded for *Liolaemus uniformis*.

**Table 1.** Scalation and morphological characteristics for the species of the *nigroviridis* group. Juvenile specimens examined are excluded. M = males; F = females. (*) Taken from Navarro and Núñez (1993). (**) Examined specimen plus Young-Downey and Moreno (1991) data. (***) Taken from Young-Downey and Moreno (1991). (****) Counted only for one specimen.

	*Liolaemus constanzae* (M = 14, F = 13)	*Liolaemus isabelae* (M = 4)	*Liolaemus juanortizi* (M = 1)	*Liolaemus lorenzmuelleri* (M = 3, F = 5)	*Liolaemus maldonadae* (M = 3)	*Liolaemus melanopleurus* (M = 2)	*Liolaemus nigroviridis* (M = 9, F = 4)	*Liolaemus uniformis* sp. n. (M = 2, F = 3)
Midbody scales	54–64	54–60	54–59**	50–62	58–64	42–56	55–64	58–62
Dorsal scales	56–67	56–67	52	44–55	48–50	40–51	45–53	56–63
Vental scales	86–96	86–97	88	86–96	83–91	91****	85–97	91–102
Nasal-rostral contact	92.6%	25%	100%	100%	100%	100%	100%	100%
Sexual dichromatism	Present	Present*	Absent***	Absent	?	?	Present	Absent
Vertebral line (males)	Present	Present/ absent	Present/ absent	Present	Absent/ inconspicuous	Absent	Absent/ inconspicuous	Absent
Maximum SVL (mm)	75,3	82,8	94.4***	88.8	85.6	70.6	73,8	89.1

*Liolaemus uniformis* differs from *Liolaemus isabelae* (Fig. 3C), because in the latter the nasal and the rostral scales are in contact only in 25% of specimens, whereas in *Liolaemus uniformis*, these scales are always in contact. Males of *Liolaemus isabelae* have black ventral coloration, a yellow dorsal color with a black vertebral line, black bars in the paravertebral fields, and a black lateral band, or some males have a completely black dorsal color; all traits that are absent in *Liolaemus uniformis*. Additionally, the southern distributional limit of *Liolaemus isabelae* in Salar de Pedernales, Atacama Region, Chile (Pincheira-Donoso and Núñez 2005) is more than 650 km north of the type locality recorded for *Liolaemus uniformis*.

*Liolaemus uniformis* resembles *Liolaemus lorenzmuelleri* (Fig. 3E) and *Liolaemus juanortizi* (Fig. 3D), species suggested as conspecific (Pincheira-Donoso and Núñez 2005). However, the dorsal scales in *Liolaemus lorenzmuelleri* and *Liolaemus juanortizi* are noticeably larger than those of *Liolaemus uniformis*, and have a distinct “ovoid” shape. *Liolaemus uniformis* has more dorsal scales (60.0 ± 2.9) than *Liolaemus lorenzmuelleri* (48.4 ± 4.2) (t = -5.4, P < 0.01). On the other hand, while only one specimen of *Liolaemus juanortizi* was examined, this one has 52 dorsal scales, which is below of the range for *Liolaemus uniformis* (Table 1). *Liolaemus uniformis* has more midbody scales (60.4 ± 1.7) than *Liolaemus lorenzmuelleri* (54.9 ± 4.5) (t = 2.6, P < 0.05) and *Liolaemus juanortizi* (56.7 ± 2.1) (t = 3.2, P < 0.05). *Liolaemus lorenzmuelleri* has a dark vertebral line and dark transversal lines running from the paravertebral fields to the flanks, whereas *Liolaemus uniformis* has no dorsal pattern. The dorsal pattern of *Liolaemus juanortizi* is very similar to *Liolaemus lorenzmuelleri*, but some specimens have a black ventral coloration, a black lateral band, and the lack of a dark vertebral line, whereas *Liolaemus uniformis* has no black ventral color or black lateral band. Additionally, the southern distributional limit of *Liolaemus lorenzmuelleri* (Embalse La Laguna, Coquimbo Region, Chile) is more than 240 km north of the type locality recorded for *Liolaemus uniformis*; and the southern distributional limit of *Liolaemus juanortizi* in Quebrada Contrabando, Atacama Region, Chile (MNHNCL collection catalog, unpublished) is more than 520 km north of the type locality recorded for *Liolaemus uniformis*.

*Liolaemus uniformis* differs from *Liolaemus melanopleurus* (a species with only three known specimens from an undetermined location, Fig. 3B) in that the latter has a blue-gray dorsal coloration (Philippi 1860) and a black lateral band running from the axilla to the midbody, features absent in *Liolaemus uniformis*. Although the type locality of *Liolaemus melanopleurus* is undetermined, the syntypes were collected by Philippi in his journey through the Atacama Desert, between the vicinities of Copiapó (27°23'S) and San Pedro de Atacama (22°54'S), more than 530 km north of the type locality recorded for *Liolaemus uniformis*.

*Liolaemus uniformis* differs from *Liolaemus maldonadae* (Fig. 3F), because males of the latter have a yellowish or reddish dorsal color with black transverse dorsal and ventral bars and black lateral band, whereas *Liolaemus uniformis* has no dorsal pattern or black transverse ventral bars. Dorsal scales in *Liolaemus maldonadae* are noticeably larger than found in *Liolaemus uniformis*, and they have an “ovoid” shape. Dorsal and ventral scale counts in *Liolaemus maldonadae* do not overlap with the same scale counts in *Liolaemus uniformis* (Table 1). Finally, the southern distributional limit of *Liolaemus maldonadae* in Los Molles (Núñez et al. 1991) is more than 150 km north of the type locality of *Liolaemus uniformis*.

**Figure 4.**
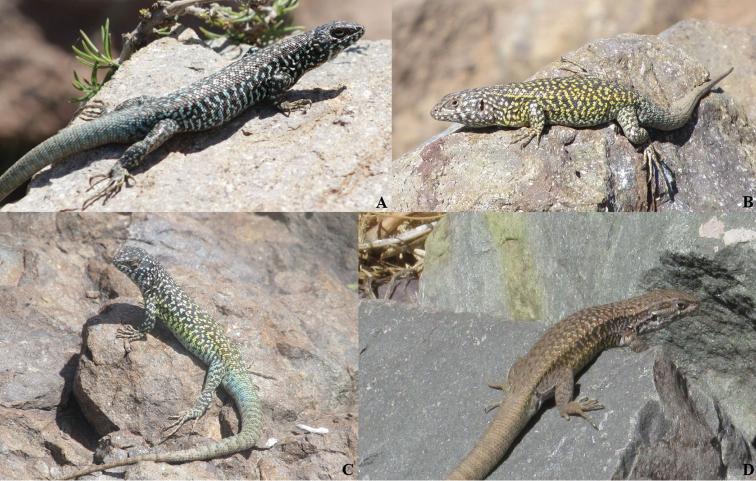
Variation in *Liolaemus nigroviridis*. **A** Male from Farellones (picture by H. Díaz) **B** Male from Carpa Mountain (picture by JTP) **C** Male from Provincia Mountain (picture by JTP) **D** Female from Juncal (picture by JTP).

**Figure 5.**
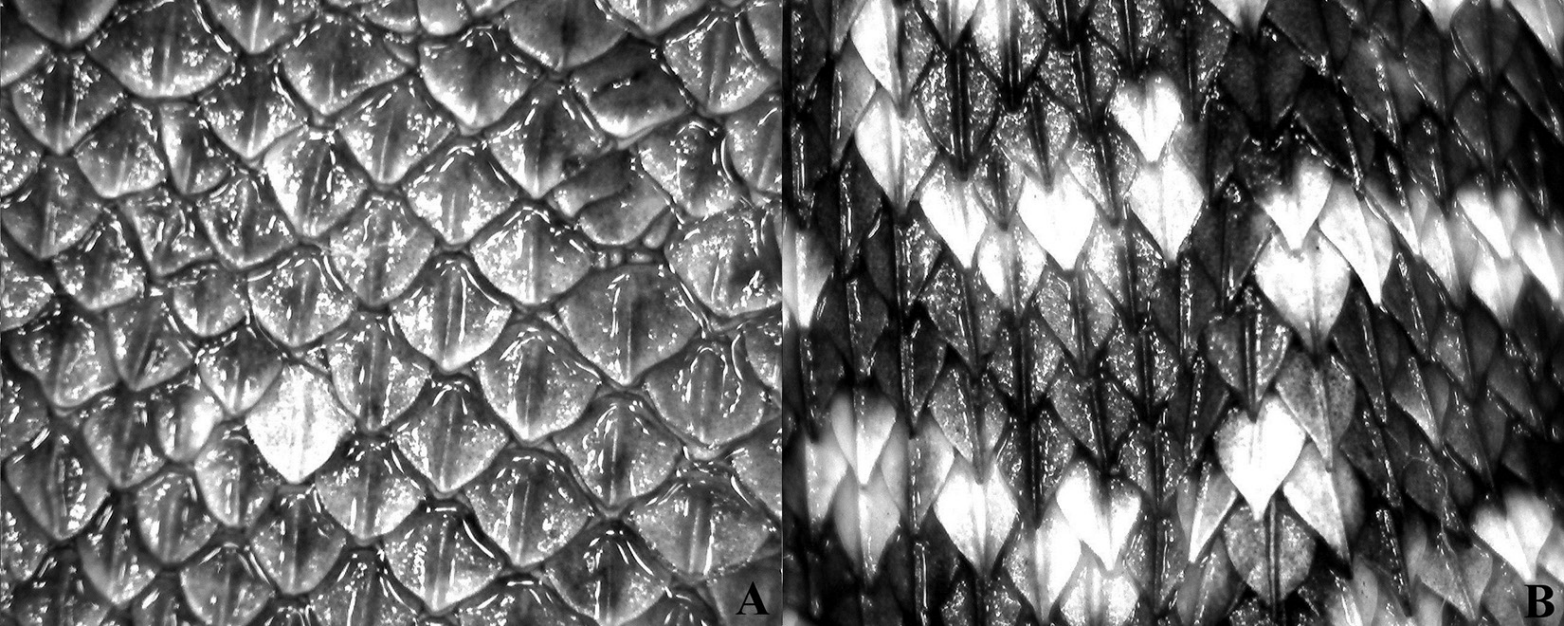
Dorsal scales, 8 mm width of view. **A** Male of *Liolaemus uniformis* sp. n. **B** *Liolaemus nigroviridis*.

*Liolaemus uniformis* is found in sympatry with *Liolaemus nigroviridis* (Fig. 4), but is larger than *Liolaemus nigroviridis* (Mann–Whitney U = 8.0, P < 0.05, Table 1). *Liolaemus uniformis* also has more dorsal scales (60.0 ± 2.9) than *Liolaemus nigroviridis* (49.4 ± 2.7) (t = 7.4, P < 0.01). *Liolaemus nigroviridis* has strongly mucronated dorsal scales, whereas *Liolaemus uniformis* has no mucrons (Fig. 5). *Liolaemus nigroviridis* has sexual dichromatism, absent in *Liolaemus uniformis*. Males of *Liolaemus nigroviridis* have a bluish or yellowish green dorsal color with black reticulation, and females have a brown dorsal color with a black lateral band, black vertebral line, and black paravertebral spots. In contrast, *Liolaemus uniformis* has a brown dorsal color without any pattern.

**Figure 6.**
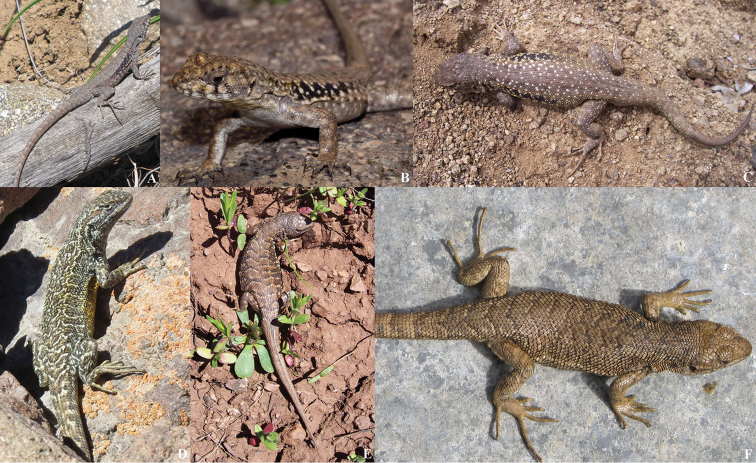
Variation in species probably confused with *Liolaemus uniformis* sp. n. **A** *Liolaemus monticola* from Salto de Apoquindo (picture by JTP) **B** *Liolaemus monticola* from La Cruz Mountain (picture by J. Abarca-Díaz) **C** *Liolaemus monticola* from Provincia Mountain (picture by JTP) **D** *Liolaemus bellii* from La Parva (JR Martini) **E** *Liolaemus bellii* from Lagunillas (picture by JTP) **F** *Liolaemus bellii* from San Ramón Mountain (picture by JTP).

Molecular data show that *Liolaemus uniformis* is not closely related to *Liolaemus monticola* (Fig. 1). Moreover, *Liolaemus monticola* is smaller (maximum SVL = 65.6 mm) than *Liolaemus uniformis* (max. SVL = 89.1 mm) (t = 3.9, P < 0.01) according to our samples, and although Pincheira-Donoso and Núñez (2005) recorded a max. SVL = 67.3 mm for *Liolaemus monticola*, the difference between both species is marked. Moreover, *Liolaemus monticola* exhibit a characteristic black lateral band between the axilla and midbody (diffuse in females), and males have white dots dispersed on the dorsum and a series of small black spots on the dorsum (Fig. 6). All these traits are absent in *Liolaemus uniformis*. The upper altitudinal limit of *Liolaemus monticola* distributions is 2000 m a.s.l. (Espinoza et al. 2004, Fuentes and Ipinza 1979), whereas *Liolaemus uniformis* has a lower altitudinal distribution limit of 2820 m a.s.l.

Molecular data show that *Liolaemus uniformis* is not closely related to *Liolaemus bellii* (Fig. 1). Moreover, *Liolaemus bellii* is smaller (maximum SVL = 80.8 mm) than *Liolaemus uniformis* (max. SVL = 89.1 mm) (t = 2.7, P < 0.05). *Liolaemus uniformis* has more midbody scales (60.4 ± 1.7) than *Liolaemus bellii* (52.9 ± 2.6) (t = 6.1, P < 0.01); more dorsal scales (60.0 ±2.9) than *Liolaemus bellii* (43.3 ±3.1) (t = 10.2, P < 0.01); and more ventral scales (96.2 ±4.8) than *Liolaemus bellii* (89.7 ±4.6) (Mann–Whitney U = 10.5, P < 0.05). Dorsal scales in *Liolaemus bellii* are strongly keeled and mucronated, whereas there are no mucrons in *Liolaemus uniformis*. Moreover, *Liolaemus bellii* exhibit a characteristic series of black dorsal “W” o “V” shaped spots (Fig. 6), whereas *Liolaemus uniformis* has no dorsal pattern.

#### Description of the holotype.

Adult male. SVL = 84.7 mm. Horizontal diameter of the eye: 4.3 mm. Subocular length: 4.5 mm. Length of the fourth supralabial: 4.1 mm. Head length (from the posterior border of the auditory meatus to the tip of the snout): 22.1 mm. Head height (distance between the two ear openings): 10.4 mm. Head width (at the level of ear openings): 15.8 mm. Neck width: 12.4 mm. Interorbital distance: 6.3 mm. Ear-eye distance: 7.5 mm. Internarine distance: 3.8 mm. Ear width: 2.5 mm. Ear height: 3.5 mm. Axillary-groin distance: 34.9 mm. Body width: 24.7 mm. Forelimb length: 25.7 mm. Hindlimb length: 46.1 mm. Length of the right hand: 10.4 mm. Length of the right foot: 22.4 mm. Tail length (not autotomized): 132.4 mm, with relation tail length/SVL = 1.56. Pentagonal rostral scale, wider (4.2 mm) than high (1.4 mm).

Two postrostrals. Four internasals. Heptagonal interparietal, with a central, small, and whitish central spot marking the position of the parietal eye. Interparietal smaller than the parietals, surrounded by seven scales. Seven scales between the interparietal and rostral. Thirteen scales between the occiput and the rostral. Orbital semicircle incomplete on the right side and complete on the left (formed by thirteen scales). Three supraoculars on the left side and four on the right. Six superciliary scales. Frontal area divided into three scales (1 posterior and 2 anterior). Preocular separated from the lorilabials by one loreal scale. Two scales between nasal and canthal. Nasal in contact with the rostral, surrounded by six scales. One row of lorilabials between the supralabials and subocular. Four lorilabials in contact with the subocular. Six supralabials, the fourth is curved upward without contacting the subocular. Four infralabials scales. Pentagonal mental scale, in contact with four scales. Four pairs of post-mental shields, the second is separated by two scales. Temporal scales smooth or slightly keeled, imbricated. Six temporal scales between the level of superciliary scales and the rictal level. Four scales on the anterior edge of the ear, which do not cover the auditory meatus. Poorly differentiated auricular scale, pentagonal and located at the upper part of the meatus. Thirty gulars between the auditory meatus. Lateral neck fold is “Y” shaped. Ventrolateral fold running from the neck to the groin. Dorsolateral fold slightly developed, running from the ear to the base of the tail. Midbody scales: 60. Dorsal scales are lanceolated, imbricated, keeled (without mucrons), with few interstitial granules. Dorsal smaller than the ventrals. Dorsal scales: 58. Ventrals scales are polymorphic (rounded, rhomboidal, pentagonal or hexagonal) smooth, imbricated, without interstitial granules. Ventrals: 91. Three precloacal pores. Supra-femoral scales lanceolate, imbricated, smooth or keeled. Infra-femoral scales lanceolate or rounded, smooth and imbricated. Supra-antebrachials scales are rounded or lanceolated, imbricated and smooth or keeled. Infra-antebrachials are rounded, imbricated and smooth. Dorsal scales of tail are pentagonal or rhomboidal, imbricated and keeled. Ventral tail scales are rounded or rhomboidal, smooth and imbricated. Lamellae of the fingers: I: 9, II: 13, III: 20, IV: 20 and V: 13. Lamellae of the toes: I: 11, II: 15, III: 21, VI: 27 and V: 17.

#### Color of the holotype in life.

The specimen is notable for its lack of pattern and uniform color. The head is brown and darker than the body. There are several white dots dispersed over the head and cheeks. The dorsum is coppery brown and has a few white-spotted scales that did not form a pattern. The subocular is brown and crossed by three white, vertical lines. The dorsal surface of the tail is light brown and without a pattern. The limbs are a dorsal-brown, similar to the dorsal surface, with white dots dispersed on the forelimbs and white transversal lines on the hindlimbs. The flanks are whitish with abundant dark brown scales. Ventrally, the hands, feet, thighs, vent, and tail are yellowish. The belly is whitish with dark dispersed spots and a dark ventral stripe. The throat is whitish with a dark thick reticulation. The precloacal pores are orange.

#### Variation in the type series.

Males are larger and more corpulent than females. In two males: SVL: 84.7–89.1 mm. Axilla-groin distance: 34.9–37.8 mm. Head length: 21.9–22.1 mm. Head width: 15.8–16.3 mm. Head height: 10.4–11.2 mm. Leg length: 45.4–46.1 mm. Arm length: 25.0–25.8 mm. Tail length: 132.4 mm in one specimen, with relation tail length/SVL = 1.56 (autotomized in the other). In three females: SVL: 67.7–73.1 mm. Axilla-groin distance: 33.1–35.7 mm. Head length: 17.8–20.0 mm. Head width: 11.8–13.3 mm. Head height: 7.5–8.3 mm. Leg length: 32.0–34.8 mm. Arm length: 19.2–21.3 mm. Tail length: 98.1 mm in one specimen, with relation tail length/SVL = 1.45 (autotomized in other).

The variation of the scalation in *Liolaemus uniformis* is as follows. Midbody scales: 58–62 (60.4 ±1.7). Dorsal scales: 56–63 (60.0 ±2.9). Ventral scales 91–102 (96.2 ±4.8). Fourth finger lamellae: 17–20 (19.0 ±1.4). Fourth toe lamellae: 25–27 (26.4 ±0.9). Supralabial scales: 6. Infralabial scales: 4–5 (4.4 ±0.6). Interparietal scale pentagonal, hexagonal or heptagonal, bordered by 5–7 scales (6.0 ±0.7). Nasal and rostral always in contact. Precloacal pores in males: 3. Precloacal pores are absent in females.

In general, all specimens have the pattern and color described for the holotype, with slight variations in shade. The male paratype has a dark brown throat. Two females have inconspicuous dark rings and an inconspicuous vertebral stripe on the dorsal surface of the tail. Also, two females have an olive hue on the snout. One female has a very inconspicuous series of dark crossbars on the paravertebral fields, which, while difficult to count, approximated eight. The juvenile has a similar pattern and color as the holotype, but it has an inconspicuous and fragmented dark vertebral line and inconspicuous dark spots on the paravertebral fields.

#### Distribution and natural history.

**Figure 7.**
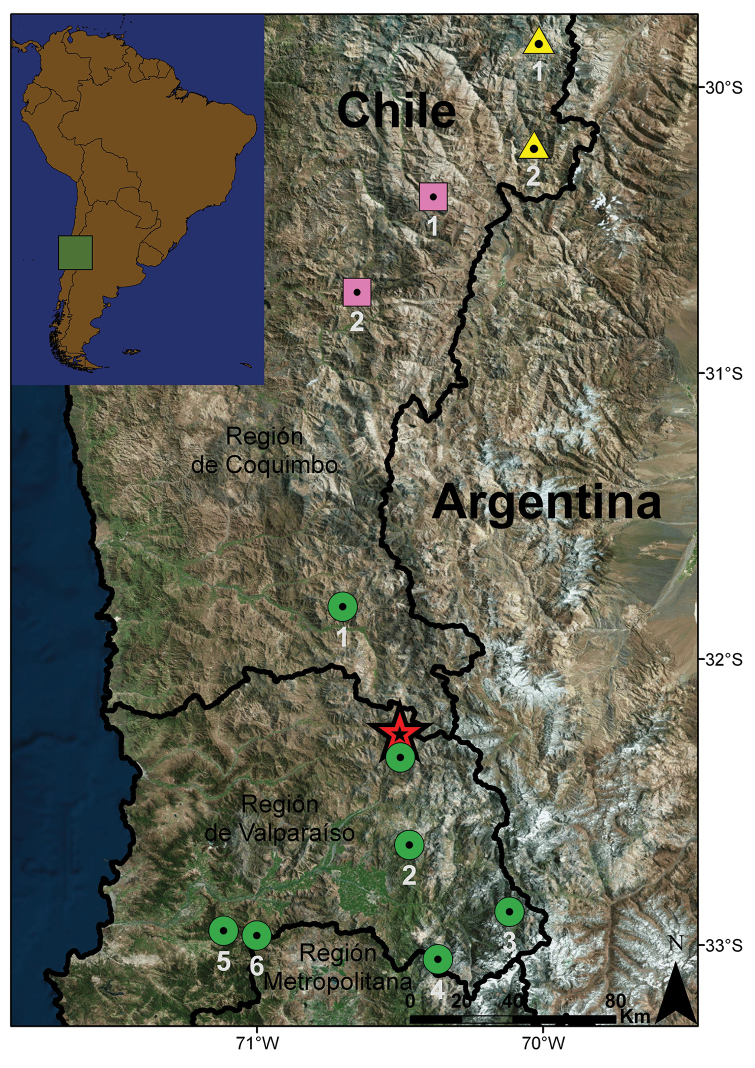
Distributional map for *Liolaemus uniformis* sp. n. along with geographically proximate species of the *nigroviridis* group. Red star: *Liolaemus uniformis* sp. n., Chepical Lagoon, type locality. Green circles: *Liolaemus nigroviridis* (1 = Manque, 2 = El Arpa, 3 = Juncal, 4 = Riecillo, 5 = La Campana, 6 = El Roble, without number = near Chepical Lagoon). Pink squares: *Liolaemus maldonadae* (1 = near Alcohuaz, 2 = Los Molles). Yellow triangles: *Liolaemus lorenzmuelleri* (1 = Baños del Toro, 2 = Embalse La Laguna).

**Figure 8.**
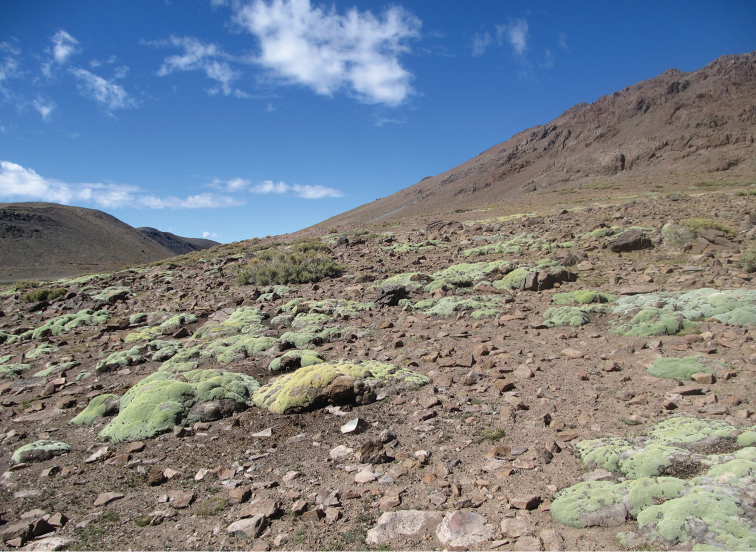
View of the type locality of *Liolaemus uniformis* sp. n., a high Andean environment.

This species is currently only known from the type locality in the surroundings of the Chepical Lagoon, approximately 30 km NE of Alicahue, in the San Felipe de Aconcagua Province, Valparaíso Region, Chile (Fig. 7). Specimens were collected on the west shore of the Chepical Lagoon (32°15'S – 70°30'W, 3050 m a.s.l.). This new species was found inhabiting rocky areas with little shrubby vegetation composed mainly of high-Andean forbs, such as *Chuquiraga oppositifolia* and *Azorella* sp. (Fig. 8). This lizard was found in abundance and was observed to have saxicolous habits. It was active between 9:00 h and 18:00 h and took refuge under rocks. Moreover, this species was found in syntopy with *Phymaturus alicahuense* Núñez, Veloso, Espejo, Veloso, Cortés & Araya 2010. Specimens were also observed at lower altitudes (32°16'S - 70°30'W, 2820 m a.s.l.) in similar environments, altitudes at which this species was found in sympatry with a few specimens of *Liolaemus nigroviridis*.

One of the collected specimens had a yellow flower inside of its mouth. An analysis of intestinal contents showed that *Liolaemus uniformis* is omnivorous; plant and Hymenoptera remains were found. A large quantity of nematodes from an unidentified species was found in the intestines. While the reproductive mode is yet unknown, at the time of sampling (December) no evidence of embryos was found but one female had several small oocytes. Comparisons with the reproductive modes of other species in the *nigroviridis* group would not be helpful as there is little available data. It is known that *Liolaemus nigroviridis* is viviparous (Donoso-Barros 1966) and *Liolaemus lorenzmuelleri* is oviparous (Cortés et al. 1995). Pincheira-Donoso and Núñez (2005) reported that *Liolaemus maldonadae* and *Liolaemus isabelae* are viviparous, but the source of this information is unclear (see Lobo et al. 2010:4) since the reproductive mode was not mentioned in the original descriptions (Navarro and Núñez 1993, Núñez et al. 1991).

## Discussion

Almost no molecular data are currently available for the *nigroviridis* group, probably due to the great difficulties of obtaining samples since all of these species inhabit high altitude mountainous areas (Pincheira-Donoso and Núñez 2005), with only *Liolaemus constanzae* (Ortiz 1975) and *Liolaemus nigroviridis* (Espinoza et al. 2004) recorded below 2000 m a.s.l. (1400 m a.s.l. and 500 m a.s.l., respectively). Moreover, most specimens from the MNHNCL and MZUC collections (the two major herpetological collections in Chile) are fixed with formaldehyde, making DNA extraction and amplification challenging (Lin et al. 2009). In regards to previous works, Torres-Pérez et al. (2009) performed three phylogenetic analysis (Bayesian inference, ML and maximum parsimony) and found that *Liolaemus nigroviridis* is the basalmost species of a clade also composed of *Liolaemus pseudolemniscatus* + *Liolaemus nigromaculatus* + *Liolaemus platei* and that this clade is closely related to *Liolaemus monticola* + *Liolaemus nitidus* clade. Our results are very similar with the *nigroviridis* and *monticola* clades as sister groups, but we did not want to include “*Liolaemus nigromaculatus*” from GenBank (Torres-Pérez et al. 2009) because the true identity of this species was only recently clarified (Troncoso-Palacios and Garín 2013) and although a specimen voucher is indicated (CUCH-3143), no locality data is provided. Since we have not seen this specimen we are not sure if it belongs to the true *Liolaemus nigromaculatus* or to *Liolaemus atacamensis*. We also did not include “*Liolaemus platei*” from GenBank (Torres-Pérez et al. 2009) because the specimen voucher (MZUC-30556) was collected in Laja Lagoon, Chile (according to MZUC Book catalog, unpublished) out of the known range for *Liolaemus platei* (Troncoso-Palacios and Marambio-Alfaro 2011), so it could be misidentified. In a recently mitochondrial ML phylogeny performed for a region spanning ND1-COI, Troncoso-Palacios et al. (2015b) found that the *Liolaemus nigroviridis* + *Liolaemus fuscus* clade is the sister group of the *monticola* clade (*Liolaemus monticola* + *Liolaemus nitidus* + *Liolaemus confusus*). This is also very similar to our result, but since there are not *Cyt-b* data for *Liolaemus fuscus*, it could not be included in the present analysis.

We recognize that one limitation to our work is that it is based in a phylogenetic analysis of only one mtDNA gene and that a wider phylogenetic DNA analysis (including nuclear genes) should be conducted in the future. This is also true for most of the 21 species of *Liolaemus* (*sensu stricto*) described in the last five years, which have been classified through different methodologies in regards to DNA comparisons. For example, three species (*Liolaemus chavin*, *Liolaemus pachacutec* and *Liolaemus wari*) include data from two mtDNA genes and shared data in GenBank (Aguilar et al. 2013). As our work, five species (*Liolaemus antumalguen*, *Liolaemus burmeisteri*, *Liolaemus cyaneinotatus*, *Liolaemus lonquimayensis* and *Liolaemus ubaghsi*) have been described with only *Cyt-b* data, and one species has been described with two mtDNA genes (*Liolaemus crandalli*). However, DNA data from all these have not been shared in GenBank or other online databases (Avila et al. 2010, 2012, 2015, Escobar-Huerta et al. 2015, Esquerré et al. 2014, Martínez et al. 2011) which does not allow the replication of the provided phylogenies or genetic distances. Two described species (Quinteros 2012, Troncoso-Palacios et al. 2015a), *Liolaemus abdalai* and *Liolaemus zabalai*, are supported in regards to DNA features by previously published phylogenetic works. Nine species (*Liolaemus aparicioi*, *Liolaemus carlosgarini*, *Liolaemus choique*, *Liolaemus chungara*, *Liolaemus nigrocoeruleus*, *Liolaemus pyriphlogos*, *Liolaemus riodamas*, *Liolaemus scorialis* and *Liolaemus smaug*) have been described without the support of molecular data (Abdala et al. 2010, Esquerré et al. 2013, Marambio-Alfaro and Troncoso-Palacios 2014, Ocampo et al. 2012, Quinteros 2012, Quinteros et al. 2014, Troncoso-Palacios et al. 2015a). Finally, one species, *Liolaemus shitan*, was described (Abdala et al. 2010) despite that no molecular differentiation was previously noted (Morando et al. 2003). No description in the last five year had included nuclear genes or more than two mtDNA genes and in most cases when DNA phylogeny is provided no data are shared in GenBank or other online databases. It is evident that *Liolaemus* researchers should put emphasis on trying to improve this situation in the future.

Although *Liolaemus uniformis* is strongly supported as a sister species of *Liolaemus nigroviridis* (pp = 1), a comprehensive phylogenetic study with more species of this group is needed. For example, *Liolaemus isabelae* was not placed within the *nigroviridis* group in a mitochondrial phylogenetic study that included one specimen (Schulte and Moreno-Roark 2010), despite that this species has been determined to be a member of this group in cladistic (Lobo 2005) and phenetic studies (Pincheira-Donoso and Núñez 2005) based on morphology. We included this species in our comparisons but for the time being, this should not be considered part of the *nigroviridis* group. Although the morphological cladistic analysis (Lobo 2005) found five apomorphies for the *nigroviridis* group (range of scale organs on postrostral scales, fourth supralabial - subocular not in contact, range of lamellae on the fourth finger, intraspecific female pattern and the relationship between the subocular length and the eye diameter), this study does not include all species currently accepted as part of the *nigroviridis* group and does not indicate the specific variation ranges of variation for these features in this group. On the other hand, the phenetic analysis of Pincheira-Donoso and Núñez (2005) does not provide supporting data for the features that were included in the matrix, so it cannot be replicated (see Lobo et al. 2010).

*Liolaemus uniformis* resembles *Liolaemus lorenzmuelleri* and *Liolaemus juanortizi* in that the three species share a similar background dorsal coloration. Although no molecular data exists to compare *Liolaemus uniformis* with these two species, we propose that the marked differences in scalation and the strongly allopatric distribution (> 240 km of separation), which is quiet considerable for lizards, support classifying *Liolaemus uniformis* as a new taxon. *Liolaemus uniformis* has probably been misidentified as *Liolaemus monticola* by Núñez et al. (2010), who noted *Liolaemus monticola* as the only lizard species to inhabit in syntopy with *Phymaturus alicahuense* (no specimen collection indicated). However, the present study found *Phymaturus alicahuense* residing at over 2900 m a.s.l, whereas the upper altitude limit for *Liolaemus monticola* is 2000 m a.s.l. (Espinoza et al. 2004, Fuentes and Ipinza 1979). Therefore, the present data indicates that the only lizards occurring in syntopy with *Phymaturus alicahuense* are *Liolaemus uniformis* and *Liolaemus nigroviridis*. Moreover, *Liolaemus uniformis* and *Liolaemus monticola* shows deep morphological and molecular differences. *Liolaemus uniformis* has probably also been confused with *Liolaemus bellii* (formerly *Liolaemus altissimus altissimus*) by Mella (2005), who found presence of the latter species in the highlands of Putaendo (no specimen collection indicated). However, a field expedition to the highlands of Putaendo by the authors of the present study found no specimens of *Liolaemus bellii*, and no additional records of *Liolaemus bellii* in this zone are known. Taking into account these details, in addition to both species having a similar background dorsal color, we think that *Liolaemus uniformis* might have been confused with *Liolaemus bellii*.

Several aspects of the *nigroviridis* group remain uncertain. For example, *Liolaemus nigroviridis* possibly contains at least two species, the nominal species from the Andean highlands and populations from Coastal highlands, formerly *Liolaemus nigroviridis campanae* (Cianferoni et al. 2013). *Liolaemus juanortizi* might be a junior synonym of *Liolaemus lorenzmuelleri* (Pincheira-Donoso and Núñez 2005), and although both are certainly very similar, it is difficult to carry out a study on this matter because the type series of *Liolaemus juanortizi* is lost (Valladares 2011) and there are very few samples of this species (Pincheira-Donoso and Núñez 2005). On the other hand, *Liolaemus melanopleurus* remains a problematic species in terms of identification as the type locality is imprecise and no additional specimens have been found in more than 100 years (Troncoso-Palacios 2012).

The present work contributes to the existing taxonomical knowledge, but the *nigroviridis* group of *Liolaemus* lizards remains poorly studied, and new samples are required to better investigate its challenging taxonomy.

## Supplementary Material

XML Treatment for Liolaemus uniformis

## Appendix I

Specimens examined. Acronyms are: Field Museum of Natural History (FMNH), Museo Nacional de Historia Natural de Chile (MNHNCL), Museo de Zoología de la Universidad de Concepción (MZUC) and Colección de Flora y Fauna, Profesor Patricio Sánchez Reyes de la Pontificia Universidad Católica de Chile (SSUC).

*Liolaemus bellii*. MNHNCL 1599. Sewell, O`Higgins Region, Chile. Elgueta M. coll. December 1982. SSUC 201–05. Casa de Piedra, Farellones, Metropolitan Region, Chile. Ferri F. coll. 12/10/2010. SSUC Re 206–09. El Colorado, Farellones, Metropolitan Region, Chile. Ferri F. coll. 13/11/2011. SSUC Re 398–404, 543. El Olivares, Metropolitan Region, Chile. Garín C. coll. SSUC Re 562–66. La Parva, Metropolitan Region, Chile. Opazo J. coll. December, 2003. SSUC Re 654, 656. Lagunillas, Metropolitan Region, Chile. Esquerré D. 15/02/2015.

*Liolaemus constanzae*. MZUC 29247, 29250–51. Toconao, Antofagasta Region. Unknown collector and date. MZUC 28763–65, 28767–69. Agua Verde (Quebrada de Taltal), Antofagasta Region. J.C. Ortiz, S. Zunino & M. Riveros colls. 10/02/1975. SSUC Re 338–347. Cuesta Barros Arana, Antofagasta Region. Ferri F. coll. 22/10/2011. SSUC Re 348. Southern Salar de Atacama, Antofagasta Region. Ferri F. coll. 24/10/2011. SSUC RE 482–83, 485, 488. El Abra, Antofagasta Region. G. Lobos & F. Torres colls. 22/11/2003. MNHNCL 1499–1500. Quebrada de Taltal, Agua Verde, 1400 m, Antofagasta Region. S. Zunino & M. Riveros colls. 10/02/1975. MNHNCL 1516–1520. Quebrada de Taltal, Agua Verde, 1480 m, Antofagasta Region. 27/09/1982. Núñez, Yáñez & Contreras colls.

*Liolaemus isabelae*. SSUC Re 157, 159, 160. El Cerrito, Salar de Pedernales, Atacama Region. F. Ferri & J. Troncoso-Palacios colls. 22/02/2012. SSUC Re 158. Montandón, Salar de Pedernales, Atacama Region. F. Ferri & J. Troncoso-Palacios colls. 22/02/2012.

*Liolaemus juanortizi*. MZUC 11782. Río Patón, Atacama Region. Cekalovic T. coll. 20/12/1963.

*Liolaemus lorenzmuelleri*. MNHNCL 2401, 2403, 2404, 2406–08. El Indio, Baños del Toro, Coquimbo Region. H. Núñez & J.C. Torres-Mura colls. 18-22/12/1992. MNHNCL 1708. La Laguna, Valle del Elqui, 3300 m, Coquimbo Region. L. Contreras coll. December, 1982. MZUC 37863–64. Valle de Los Helados, Copiapó, Atacama Region. Asociación de Andinistas de Atacama colls. 13/04/1984.

*Liolaemus maldonadae*. SSUC Re 304, 305, 560. Quebrada Los Piuquenes, Interior de Alcohuaz, Paihuano, Río Claro, Coquimbo Region. Troncoso-Palacios, J., F. Lobo, A. Laspiur & J.C. Acosta Colls. 10/02/2011.

*Liolaemus melanopleurus*. MNHNCL 1646 (2 specimens). Atacama. R.A. Philippi col. FMNH 9969 (only digital photographs). Atacama. R.A. Philippi coll.

*Liolaemus monticola*. SSUC Re 372–79. Camino a Farellones, Curva 20, Metropolitan Region, Chile. Ferri F. coll. 15/03/2012.

*Liolaemus nigroviridis*. MNHNCL 214–215. San Ramón, 3000 m, Metropolitan Region. H. Núñez coll. February, 1979. SSUC Re 016. El Yeso, Metropolitan Region. C. Garín coll. 01/04/2004. SSUC Re 190–200. Farellones, Casa de Piedra, Camino a Valle Nevado, Metropolitan Region. F. Ferri coll. 12/10/2010.

*Liolaemus uniformis*. SSUC Re 674–79. West shore of the Chepical Lagoon, approximately 30 km NE Alicahue, San Felipe de Aconcagua Province, Valparaíso Region, Chile. J. Troncoso-Palacios & E. Alfaro. December, 2012.

## Appendix II

Specimens used for phylogenetic analysis.

mtDNA sequences obtained in this study. *Liolaemus uniformis* sp. n.: SSUC Re 674, KU095836. SSUC Re 677 KU095837. *Liolaemus nitidus*: SSUC Re 298, Dunas de Ritoqui, Valparaíso Region, Chile. KU095835. *Liolaemus confusus*: SSUC Re 356, Cerro Robles Riscos de Jote, O`Higgins Region, Chile. KU095832. *Liolaemus curicensis*: SSUC Re 253, Termas del Flaco, O`Higgins, Chile. KU095833. *Liolaemus kuhlmanni*: SSUC Re 285, Termas del Flaco, O`Higgins, Chile. KU095834. *Liolaemus bellii*: SSUC Re 208, El Colorado, Farellones, Metropolitan Region. KU095830. *Liolaemus bellii*: SSUC Re 209, El Colorado, Farellones, Metropolitan Region. KU095831.

mtDNA sequences obtained from GenBank. *Liolaemus nigroviridis*: Farellones. KC313199, KC313202, KC313203, KC313204, KC313205, KC313206, KC313208, KC313211, KC313207, KC313210, KC313209. *Liolaemus monticola*: Yerba Loca AY850619, Alfalfal AY850616, Maipú AY851724, Cuesta Chacabuco AY851718, Quebrada Alvarado AY851726, Colorado Norte AY851713, Cabrería AY851708, Rocín AY851710. *Liolaemus tenuis*: Termas de Chillán DQ989790. *Liolaemus abdalai*: Valle Chimehuin JN410525. *Liolaemus alticolor*: Huancarani KF923660. Santa Ana KF923659. *Liolaemus austromendocinus*: Nihuil AY173838. *Liolaemus buergeri*: El Planchón KJ494079, KJ494070, KJ494080. *Liolaemus capillitas*: Ruta Provincial AY173844. *Liolaemus chiliensis*: Termas de Chillan DQ989785, Las Trancas EU649245. *Liolaemus cyanogaster*: Tucapel DQ989786. *Liolaemus dicktracy*: Alto del Carrizal AY367816. *Liolaemus elongatus*: Esquel AY173801, Gobernador Costa AY173818, Los Manantiales AY173826, Laguna Blanca AY173855, Pampa de Lonco Luan AY173827, Las Ardillas AY173852. *Liolaemus gununakuna*: La Amarga AY367807, AY173859. *Liolaemus incaicus*: Urco KF923658, Lucre KF923657. *Liolaemus kriegi*: all from Río Negro Province AY173802, KJ494012, KJ494150, KJ494190, AY173814, KJ494155, KJ494191, KJ494188. *Liolaemus neuquensis*: Primeros Pinos AY173828. *Liolaemus parvus*: Quebrada Honda AY173836. *Liolaemus petrophilus*: El Cuy AY173796, Los Menucos JN847211, Ingeniero Jacobacci JN847103. *Liolaemus pictus*: San Carlos de Bariloche AY173795. *Liolaemus punmahuida*: Volcán Tromen AY173824. *Liolaemus ramirezae*: E Amaicha del Valle JN410520. *Liolaemus robertmertensi*: Tinogasta DQ989769. *Liolaemus saxatilis*: Achiras JN410553, Río Cuarto JN410527. *Liolaemus smaug*: Las Leñas AY173832, Mallines Colgados AY173830. *Liolaemus talampaya*: Las Yeguas River AY173797. *Liolaemus tregenzai*: all from Termas de Copahue AY367817, KJ494036, KJ494230, KJ494040, KJ494039, KJ494037, KJ494038. *Liolaemus tulkas*: Quebrada Las Angosturas AY367813. *Liolaemus umbrifer*: Quebrada de Randolfo AY367814. *Liolaemus villaricensis*: Volcán Villarrica AY850629, AY730671. *Liolaemus zabalai*: all from Biobío Region KJ494059, KJ494056, KJ494057, KJ494086, KJ494074, KJ494085. *Phymaturus vociferator*: Laguna del Laja JX969016. *Phymaturus felixi*: Paso de Indios JX969044.
